# Combinations of two imaging parameters to improve bone mineral density (BMD) assessment in patients with lumbar degenerative diseases

**DOI:** 10.1186/s12891-023-06888-8

**Published:** 2023-09-21

**Authors:** Wenshuai Li, Houze Zhu, Hongsen Tian, Tong Tong, Zijian Hua, Xuan Zhao, Yong Shen, Linfeng Wang

**Affiliations:** 1 Department of Orthopaedic Surgery, The Third Hospital of Hebei Medical University, 139 Ziqiang Street, Shijiazhuang, 050051 Hebei People’s Republic of China; 2 The Key Laboratory of Orthopedic Biomechanics of Hebei Province, The Third Hospital of Hebei Medical University, Shijiazhuang, 050051 People’s Republic of China

**Keywords:** Osteoporosis, Hounsfield units, Vertebral bone quality, Lumbar degenerative disease

## Abstract

**Purpose:**

To explore whether combining the Hounsfield unit (HU) values and vertebral bone quality (VBQ) scores can improve the BMD assessment in patients with lumbar degenerative diseases.

**Methods:**

The HU values were measured by CT image, and VBQ scores were calculated by lumbar MRI image. The correlations of the opportunistic imaging parameters to the lowest T-scores were analyzed. Receiver-operating characteristic curve (ROC) analysis was used to evaluate the accuracy in detecting osteoporosis. Finally, the specificity and sensitivity of different combined methods of the HU values and VBQ scores in the diagnosis of osteoporosis were compared.

**Results:**

Patients with osteoporosis had the lowest HU values and the highest VBQ scores. The correlation coefficients between the VBQ scores and the T-scores were smaller than HU values (L1 HU value: 0.702; average HU value:0.700; L1 VBQ score: -0.413; VBQ score: -0.386). The areas under the curve (AUCs) of the HU values were greater than those of the VBQ scores, and the AUCs of the L1 VBQ score were similar to the VBQ score (L1 HU value: 0.850; average HU value:0.857; L1 VBQ score: 0.704; VBQ score: 0.673). When combining the two imaging parameters in series, the specificity of the detection of osteoporosis was improved (L1 HU value and L1 VBQ score: 87.3%; Average HU value and VBQ score: 85.9%). When combining the two imaging parameters in parallel, the sensitivity of the detection of osteoporosis was improved (L1 HU value or L1 VBQ score: 88.1%; Average HU value or VBQ score: 91.5%).

**Conclusions:**

Combinations of the HU values and VBQ scores could improve the diagnostic performance of osteoporosis. In addition, considering the same diagnostic performance but easier measurement, parameters at the single-segment level were recommended to assist in the diagnosis of osteoporosis.

## Introduction

Osteoporosis is defined as low bone mass and micro-architectural deterioration of bone tissue with consequent increases in bone fragility and susceptibility to fracture [1]. As the population ages, osteoporosis has become a common disease. Especially in patients requiring spinal surgery, bone mineral density (BMD) assessment is critical. Osteoporosis is one of the important risk factors for complications such as fixation failure, screw loosening, and pseudarthrosis after spinal surgery [2–4]. Dual energy x-ray absorptiometry (DXA) is presently used as the gold standard method for assessing BMD [5]. WHO diagnostic criteria for osteoporosis are often used clinically [4]: osteoporosis (the lowest T-score ≤ − 2.5), osteopenia (− 2.5 < the lowest T-score < − 1), and normal BMD (the lowest T-score ≥ − 1). However, DXA sometimes may not reflect the vertebral cancellous BMD well because of degenerative arthritis, osteophyte formation, and spinal sclerosis [6–8]. To more accurately measure the vertebral cancellous BMD, several tools that assist DXA have been widely studied [9–12].

The Hounsfield unit (HU) value obtained by computed tomography (CT) scanning was considered a useful technique for the assessment of lumbar BMD, and the corresponding threshold has been established [9–11]. Schreiber et al. [13] initially found that the HU value was significantly correlated with BMD and T-score, and also significantly correlated with compressive strength. Subsequently, Pickhardt et al. [14] used abdominal CT to perform opportunistic screening for osteoporosis, further improved a study involving 1867 samples, and established corresponding thresholds. When the HU value is close to 110, it is considered that the screening of osteoporosis has good specificity. After that, a large number of studies proved the correlation between lumbar HU value and BMD and also obtained similar conclusions. In addition, some studies have also found that the HU value can predict osteoporosis-related complications [15, 16].

Another novel technique for assessing bone quality is the vertebral bone quality (VBQ) score, which uses non-contrast, T1-weighted lumbar spine MRI and has a good diagnostic ability of osteoporosis [12, 17]. When the T-score was used as a criterion, the VBQ score was about 80% accurate in determining osteoporosis and the threshold of VBQ score is close to 3.0 [18]. Subsequently, a study has shown that the VBQ score is an independent predictor of fragility fractures [19]. More recently, the VBQ score is an effective indicator of bone quality in patients with osteoporotic compression fractures [20]. In addition, the VBQ score has been shown to have moderate to excellent intra-rater reliability (ICC:0.667–0.957) and good inter-rater reliability (ICC: 0.818), which makes the method easy to generalize [21].

A common benefit of the HU value and VBQ score is that they can be used to measure the region of interest (ROI), so the region that affects the measurement of cancellous bone in the vertebral body can be avoided. Because lumbar CT and MRI are often routine examinations for patients undergoing lumbar surgery, another common benefit of the HU value and VBQ score is the use of opportunistic imaging to provide meaningful data on bone mass that avoids additional financial burden. Although these two parameters are readily available to surgeons in clinical practice, there is no research on which one is better and how to use them properly when both parameters coexist.

Our objective was to study the characteristics of the opportunistic imaging parameters, the HU values and VBQ scores, for BMD assessment in the same patient. In this study, we not only analyzed the similarities and differences between them but also provided data support for the rational use of them in clinical practice.

## Materials and methods

### Patients

The study was approved by the Ethics Committee of our hospital (K2022-115-1). Because it was a retrospective study, signing informed consent was waived. We reviewed patients with degenerative lumbar diseases at the spinal department of our hospital from January 1, 2019, to July 1, 2021. Inclusion criteria: patients with degenerative lumbar diseases who received lumbar CT, MRI, and DXA scan at the same time within 1 month before surgery in our hospital. The exclusion criteria were: (1) Severe lumbar degeneration, such as at least 3 vertebral osteophytes with severe hyperplasia or at least 3 discs with grade 4 degeneration or narrowing of at least 3 adjacent facet joints (< 1 mm) with large osteophytes [10]; (2) a history of lumbar surgery; (3) spinal infection, tumor, or metabolic disease; (4) anatomical identification is difficult to identify for radiometry. In the end, a total of 130 patients were included in the study.

### BMD evaluation

T-scores were measured by DXA (Discover A densitometers, Hologic Inc, Bedford, MA, USA). WHO criteria for diagnosing osteoporosis were applied [5]: osteoporosis (the lowest T-score ≤ − 2.5), osteopenia (− 2.5 < the lowest T-score < − 1), and normal BMD (the lowest T-score ≥ − 1).

### HU values evaluation

**Fig. 1:**
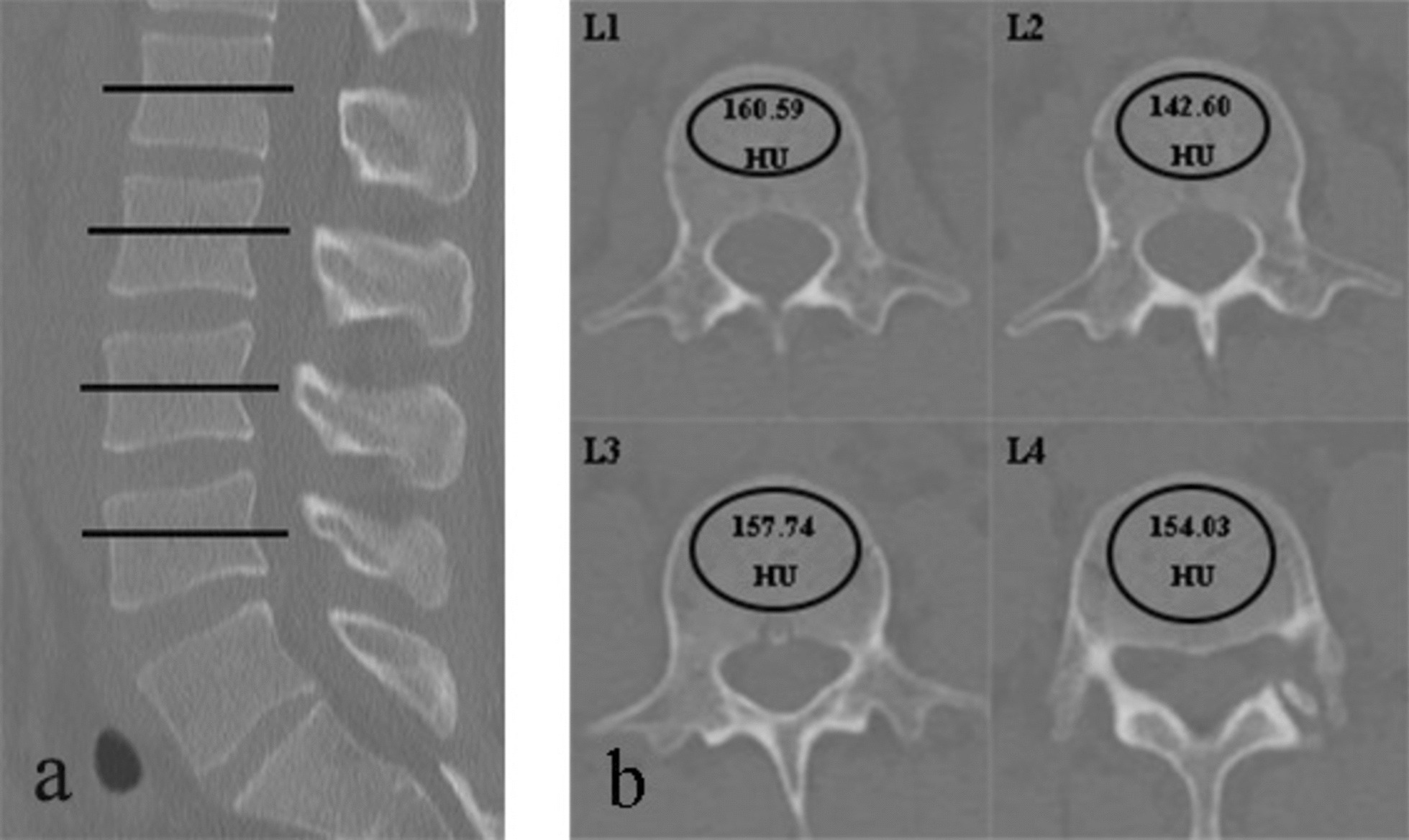
HU values determined using region of interest (ROI) were shown. Figure 1 A shows the axial plane of ROI in the L1-L4. Figure 1B shows PACS software automatically calculates the HU values

As previous protocol [10], PACS was used to calculate HU values. Briefly, HU values were measured by placing the elliptic ROI in an axial mid-body image in L1-L4 (Fig. 1). Include as many trabeculae as possible in ROI and avoid cortical bone and heterogeneous areas such as bone islands, and compressed bone.

### VBQ scores evaluation

**Fig. 2:**
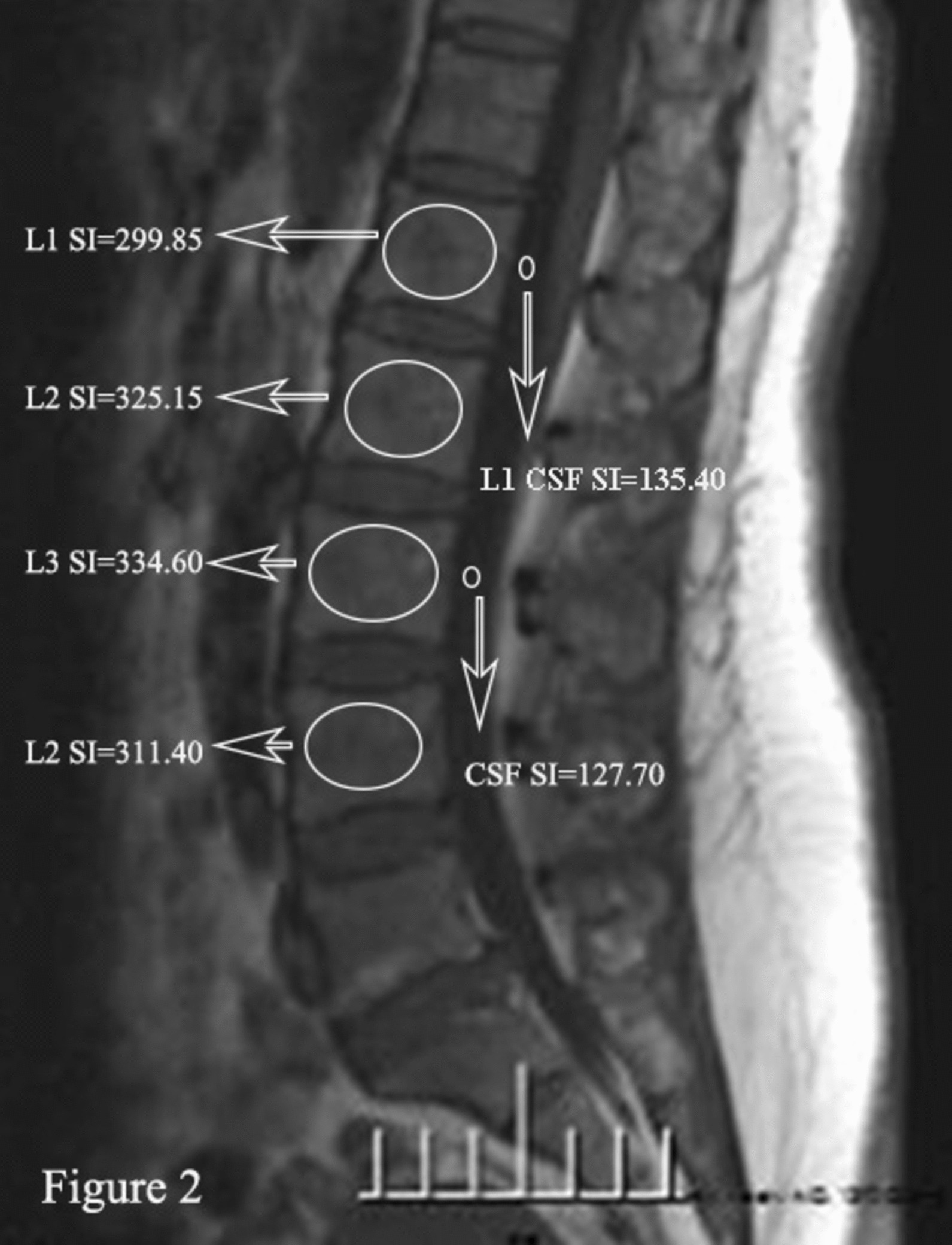
Non − contrast-enhanced T1-weighted MRI image shows the determination of signal intensity (SI) of L1-L4 and SI of cerebrospinal fluid (CSF) at the L1 level and L3 level using regions of interest (ROIs)

As previously described [12, 17], the VBQ scores were assessed using lumbar non-contrast, T1-weighted MRI. Firstly, the ROI was placed in a mid-sagittal section to measure the signal intensity (SI) of the vertebral body in L1-L4 (Fig. 2). In patients with mid-sagittal abnormalities such as hemangioma or venous plexus, parasagittal slices were used to reflect bone quality. If the entire vertebral body is abnormal, the vertebral body is excluded. The median SI of L1-L4 was then divided by the SI of cerebrospinal fluid (CSF) at the L3 level to obtain the VBQ score (Fig. 2). If the CSF of the L3 space is completely blocked, the ROI of the CSF is placed at the L2 level.

$$\mathrm{VBQ}\;\mathrm{score}\;=\;\frac{\mathrm{Median}\left({\mathrm{SI}}_{L1-L4}\right)}{{\mathrm{SI}}_{L3\;CSF}}$$

The L1 VBQ score was calculated as the ratio of the SIs in the L1 and SIs in the CSF at the L1 level [17].

$$\text{L}1\ \text{V}\text{B}\text{Q}\ \text{s}\text{c}\text{o}\text{r}\text{e}=\frac{{\text{S}\text{I}}_{L1}}{{\text{S}\text{I}}_{L1\ CSF}}$$

All HU values and VBQ scores were measured by two independent observers and averaged for statistical analysis. To test inter-rater reliability, an observer randomly selected 20 patients to take the same measurement three weeks later.

### Statistical analysis

SPSS 27 (SPSS, USA) software was used for statistical analysis. Shapiro-Wilk tests and F tests were used for normality and homogeneity of the data, respectively. The intraclass correlation coefficient (ICC) was calculated to test inter-rater and intra-rater reliability (0 represents no agreement and 1 represents perfect agreement). One-way analysis of variance (ANOVA) tests were used to compare the variables that conform to a positive distribution with homogeneous variance among the three groups. Kruskal Wallis tests were used to compare the variables that do not conform to a normal distribution or have non-homogeneous variances. Quantitative data were analyzed with the chi-square or Fisher’s exact test. The correlations between the lowest T-score and HU values or VBQ scores were evaluated by the Pearson correlation coefficient. For the correlation coefficient (r), r ≤ 0.3 represents poor correlation, 0.3 < r ≤ 0.6 represents moderate correlation, 0.6 < r ≤ 0.8 represents high correlation, and r > 0.8 represents extremely high correlation. Receiver-operating characteristic curve (ROC) analysis was used to evaluate the accuracy of HU values and VBQ scores in detecting osteoporosis, and the corresponding threshold was established by the Youden index [17]. The specificity and sensitivity of different combined methods of HU values and VBQ scores in the diagnosis of osteoporosis were compared.

## Results

Firstly, patients were grouped according to the BMD, and the demographic characteristics of different groups were compared. There were 130 patients in the study, including 50 males and 80 females. Normal BMD was detected in 20 patients, osteopenia in 51 patients, and osteoporosis in 59 patients. The average age was 59.4 years and patients with osteopenia and osteoporosis were older than the normal BMD group (55.3, 57.9 vs. 62.0 years, *p* < 0.05). The other characteristics of these patients were also summarized (Table 1).

**Table 1:** Demographic characteristics among all three bone groups

Characteristics	All	Normal BMD	Osteopenia	Osteoporosis	*P* value
Number	130 (100%)	20 (100%)	51 (100%)	59 (100%)	—
Age(years)	59.4 ± 9.6	55.3 ± 8.6	57.9 ± 11.6	62.0 ± 7.0	0.009
female	80 (61.5%)	14 (70.0%)	30 (58.8%)	36 (61.0%)	0.680
BMI (kg/m^2^)	25.69 ± 3.24	27.00 ± 2.93	25.54 ± 3.40	25.36 ± 3.12	0.136
Current smoker	26 (20%)	3 (15.0%)	7 (13.7%)	16 (27.1%)	0.179
Hyperlipidemia	65 (50%)	10 (50.0%)	23 (45.1%)	32 (54.2%)	0.633
Diabetes	23(17.7%)	4 (20.0%)	10 (19.6%)	9 (15.3%)	0.802
Glucocorticoid use	18 (13.8%)	0 (0.0%)	5 (9.8%)	10 (16.9%)	0.108

**Fig. 3:**
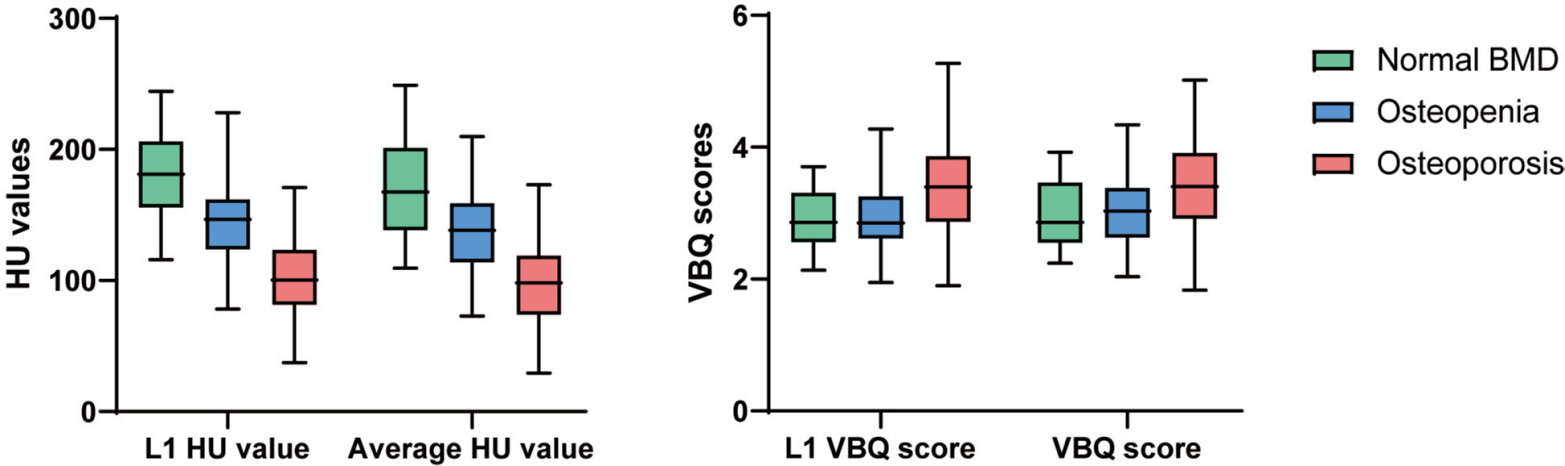
The distribution of HU values and VBQ scores in different BMD was shown. The line in the box plot represents the median, and the top to bottom of the box is the range of quartiles

To observe whether the HU values and VBQ scores can reflect BMD of different parts of different groups, the characteristics of the T-scores, HU values and VBQ scores were described. The average lowest T-score was − 2.21 and patients with osteoporosis and osteopenia were lower than the normal BMD group (-3.19, -1.80 vs. -0.36, *p* < 0.001). The same trend was observed for the T-scores of the total hip, femoral neck, and L1-L4 (Table 3). The ICCs for both intra-rater and inter-rater reliability of the HU values and VBQ scores were above 0.8 (Table 2). Both L1 HU value and average HU value were lowest in patients with osteoporosis, followed by patients with osteopenia and those with normal BMD (L1 HU value: 103.2 vs. 147.1 vs. 180.5, *p* < 0.001; average HU value: 96.2 vs. 139.7 vs. 169.6, *p* < 0.001) (Table 3). In addition, patients with osteoporosis had the highest VBQ scores among all three bone groups (L1 VBQ score: 3.40 vs. 2.96 vs. 2.91, *p* < 0.001; VBQ score: 3.42 vs. 3.06 vs. 2.98, *p* = 0.003). The box plots of HU values and VBQ scores according to the WHO diagnostic criteria for BMD are shown in Fig. 3.

**Table 2:** The ICCs of HU values and VBQ scores

	Inter-rater	Intra-rater
**HU values**		
L1 HU value	0.960	0.955
L2 HU value	0.934	0.916
L3 HU value	0.952	0.935
L4 HU value	0.942	0.900
**VBQ scores**		
L1 VBQ score	0.836	0.885
VBQ score	0.829	0.853

**Table 3:** Bone assessment using DXA, CT, and MRI among all three bone groups

	All	Normal BMD	Osteopenia	Osteoporosis	*P* value
**DXA**					
Lowest T-score	-2.21 ± 1.13	-0.36 ± 0.56	-1.80 ± 0.41	-3.19 ± 0.54	<0.001
Total hip T-Score	-0.89 ± 0.98	0.42 ± 0.65	-0.62 ± 0.61	-1.55 ± 0.76	<0.001
Femoral neck T-score	-1.63 ± 1.01	-0.27 ± 0.58	-1.33 ± 0.59	-2.35 ± 0.77	<0.001
L1 T-score	-1.38 ± 1.29	0.32 ± 0.68	-1.05 ± 0.97	-2.24 ± 0.94	<0.001
L2 T-score	-1.20 ± 1.48	0.67 ± 0.99	-0.80 ± 1.14	-2.16 ± 1.06	<0.001
L3 T-score	-1.08 ± 1.56	0.83 ± 0.90	-0.66 ± 1.22	-2.11 ± 1.16	<0.001
L4 T-score	-0.29 ± 1.77	1.90 ± 1.16	0.05 ± 1.32	-1.35 ± 1.45	<0.001
**HU values**					
L1 HU value	132.3 ± 44.6	180.5 ± 35.9	147.1 ± 36.9	103.2 ± 31.1	<0.001
Average HU value	124.5 ± 43.7	169.6 ± 37.8	139.7 ± 35.4	96.2 ± 31.3	<0.001
**VBQ scores**					
L1 VBQ score	3.16 ± 0.63	2.91 ± 0.36	2.96 ± 0.55	3.40 ± 0.68	<0.001
VBQ score	3.21 ± 0.64	2.98 ± 0.51	3.06 ± 0.55	3.42 ± 0.70	0.003

**Fig. 4:**
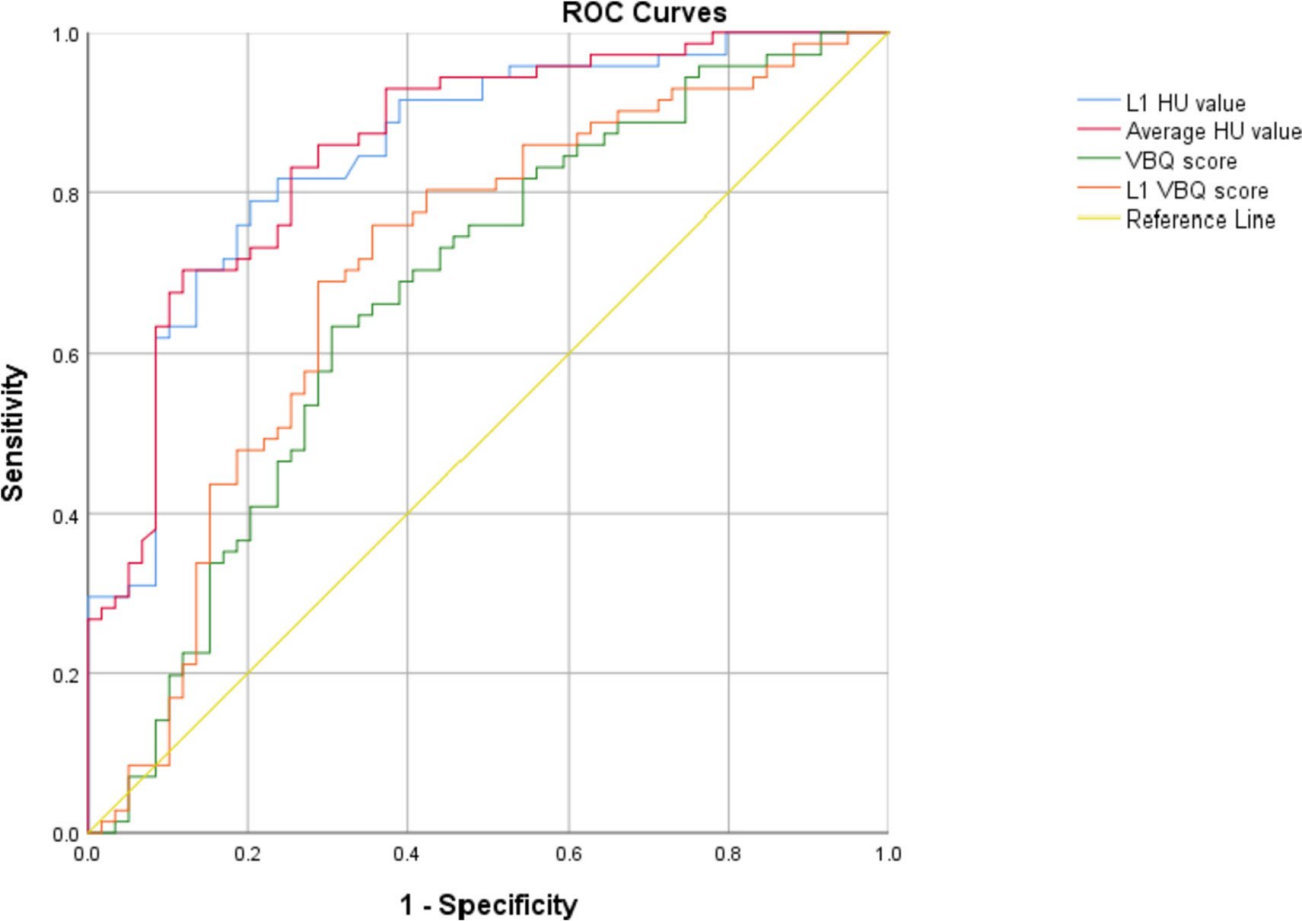
Receiver-operating characteristic curve (ROC) analysis was used to evaluate the performance of HU value and VBQ score in distinguishing osteoporosis

Subsequently, in order to make the two parameters more reasonable in clinical application, we analyzed the characteristics of the HU values and VBQ scores for screening osteoporosis. The correlation coefficient between VBQ scores and the lowest T-score was smaller than HU values (L1 VBQ score: *r* = -0.413; VBQ score: *r* = -0.386; L1 HU value: *r* = 0.702; HU value: *r* = 0.700) (Table 4). According to the WHO criteria for the diagnosis of osteoporosis, ROC curves of HU values and VBQ scores were drawn (Fig. 4), and relevant thresholds were also established (Table 5). The areas under the curves (AUCs) were larger for the HU values than for VBQ scores (L1 HU value: 0.850 vs. L1 VBQ score: 0.704, *p* = 0.002; average HU value:0.857 vs. VBQ score: 0.673, *p* < 0.001). In addition, the AUCs of the L1 HU value and L1 VBQ score were comparable to the average HU value and VBQ scores.

**Table 4:** Pearson correlations between the lowest T-score and HU value or VBQ score

	lowest T-score	*p* value
**HU values**		
L1 HU value	0.702	< 0.001
Average HU value	0.700	< 0.001
**VBQ scores**		
L1 VBQ score	-0.413	< 0.001
VBQ score	-0.386	< 0.001

**Table 5:** Diagnostic thresholds of HU value or VBQ score detecting osteoporosis

	Thresholds	Youden index	AUC	*P* value
**HU values**				
L1 HU value	124.7	0.585	0.850	< 0.001
Average HU value	126.3	0.586	0.857	< 0.001
**VBQ scores**				
L1 VBQ score	3.26	0.405	0.704	< 0.001
VBQ score	3.20	0.329	0.673	0.001

Finally, to verify whether combining the two parameters could enhance screening for osteoporosis. The sensitivity and specificity of two different combinations of opportunistic imaging parameters in the diagnosis of osteoporosis were also analyzed. The series method means that the threshold of both parameters must be met, while the parallel method means that only one of the parameters needs to be met. When combining the L1 HU value and L1 VBQ score in series and parallel, the specificity and sensitivity of the diagnosis of osteoporosis were improved, respectively. (Single L1 HU value: specificity = 78.9%, sensitivity = 79.7%; Single L1 VBQ score: specificity = 64.4%, sensitivity = 76.1%; L1 HU value and L1 VBQ score: specificity = 87.3%, sensitivity = 55.9%; L1 HU value or L1 VBQ score: specificity = 67.6%, sensitivity = 88.1%). Similar results were shown when the average HU and VBQ scores were combined in series and parallel (Table 6).

**Table 6:** Diagnostic accuracy of the HU value and VBQ score detecting osteoporosis

	Specificity	Sensitivity
L1 HU value	78.9%	79.7%
L1 VBQ score	64.4%	76.1%
Average HU value	70.4%	88.1%
VBQ score	63.4%	69.5%
L1 HU value and L1 VBQ score	87.3%	55.9%
L1 HU value or L1 VBQ score	67.6%	88.1%
Average HU value and VBQ score	85.9%	66.1%
Average HU value or VBQ score	52.1%	91.5%

## Discussion

In this study, both opportunistic imaging parameters could be used as tools to assess BMD in patients with degenerative lumbar diseases. Interestingly, we found for the first time that the results of diagnosing osteoporosis of the HU values and VBQ scores did not completely overlap, and the use of these parameters in series and parallel could effectively improve the specificity and sensitivity of the screening of osteoporosis. In addition, the L1 HU value and L1 VBQ score may be suitable for clinical use because they are simpler to measure while ensuring diagnostic performance similar to the average HU value and VBQ score.

CT-based HU value indicates the density of the measured object [13]. Many studies had shown a good correlation between HU value and T-score, and HU value could be used as a complementary tool to assess bone mass [9–11, 13, 22, 23]. Currently, the L1 HU value was usually used as an indicator for the diagnosis of osteoporosis [24]. This method did not require additional results of HU values of all vertebral bodies and could ensure good diagnostic efficiency. Similarly, the M-score was the first MRI-based observation to use adipose tissue with a high T1 signal and to assess fat infiltration in cancellous bone by measuring the T1 signal in the vertebral body [25], which has been proven to be useful for evaluating bone mass [26]. However, M-Score is limited because it requires measurements to be taken using the same MRI machine [26]. Recently, using the principle of M-score and avoiding its defects, the VBQ score was developed [12], which was standardized with L3 space CSF SI as a reference value and could be compared between patients measured by different MRI machines. The VBQ score is also considered to be a predictor of fragile vertebral fractures [19]. Lumbar CT and MRI are both routine examinations for patients with degenerative lumbar diseases undergoing lumbar surgery. However, previous studies only focused on the diagnostic value of a single imaging parameter for osteoporosis. Therefore, for more accurate measurement of BMD in patients undergoing lumbar surgery, it is worth considering how to apply these two parameters properly when they exist at the same time.

The establishment of HU value and VBQ score thresholds requires a gold standard as a reference. As we all know, DXA is the gold standard for diagnosing osteoporosis. However, due to the severity of spinal degeneration, DXA could not reflect the actual BMD of the cancellous bone of the vertebral body [6–8]. Therefore, we excluded patients with severe lumbar degeneration to ensure the accuracy of the “gold standard” and to obtain a more accurate threshold. According to the WHO criteria for the diagnosis of osteoporosis, the patients were divided into a normal BMD group, an osteopenia group, and an osteoporosis group. It is well known that aging is an important factor in osteoporosis. In this study, osteoporosis patients also had the lowest age. HU values were significantly different among the three groups, with the lowest HU values in patients with osteoporosis and the highest HU values in patients with normal BMD. This was consistent with previous studies, as HU values can reflect tissue density in ROI [9–11, 13, 22, 23]. In addition, the difference of the VBQ scores among the three groups was statistically significant. Patients with osteoporosis had the highest MRI-related parameters, also consistent with previous studies [12, 17]. In the correlation analysis, HU values were highly correlated with the lowest T-score, and VBQ scores were moderately correlated with the lowest T-score. These implied that HU values may be better than VBQ scores in assessing BMD, but it still needs further verification by ROC analysis.

Next, we analyzed the diagnostic performance of each imaging parameter in the diagnosis of osteoporosis by ROC curve. Similar to the correlation coefficient results, the AUCs of the HU values in the diagnosis of osteoporosis were greater than that of VBQ scores, so the diagnostic performance of HU values was better than those of VBQ scores. This may be because the HU value could directly measure bone and reflect bone mass [13], while the VBQ score can indirectly reflect bone quality by measuring fat content [12]. It should be mentioned that the VBQ scores also have certain diagnostic effects for osteoporosis and therefore cannot be ignored.

In addition, we found that the performance of LI HU value and L1 VBQ score was comparable to the average HU value and VBQ score in the diagnosis of osteoporosis (L1 HU value: AUC = 0.850; average HU value: AUC = 0.857; L1 VBQ score: AUC = 0.704; VBQ score: AUC = 0.673). This meant that not only L1 HU can measure BMD, but also the L1 VBQ score could measure BMD. Therefore, considering the same performance but easier measurement, the LI HU value and L1 VBQ score are recommended as tools to assist in the diagnosis of osteoporosis.

Finally, both imaging parameters are readily available in patients with lumbar degenerative disease requiring surgery. Considering that these two parameters reflect BMD from the perspective of bone and fat respectively, we hypothesized that the accuracy of the diagnosis of osteoporosis could be improved by combining the two parameters. As we guessed, the results showed that the specificity of the diagnosis of osteoporosis could be significantly improved when the two imaging parameters were in series and the sensitivity can be improved when they are in parallel. These indicated that the results of HU values in the diagnosis of osteoporosis and VBQ scores did not completely overlap, and to some extent, the two could complement each other. Different from the previous studies that only used a single indicator, the HU value or VBQ score, we proposed to combine the two indicators to assist the diagnosis of osteoporosis to improve the specificity and sensitivity, and provided data support. For most healthy people, CT and MRI are not routine examinations in the physical examination. However, this method may be more suitable for some patients requiring lumbar surgery because lumbar CT and MRI are often preoperative routine examinations in such patients, and the HU values and VBQ scores can be obtained opportunistically at no additional cost.

It is important to mention that some complications related to osteoporosis, such as vertebral compression fractures, are usually due to the insufficient mechanical strength of the bone itself. However, this strength is mainly due to the vertical direction of the bone under the force. Unfortunately, current techniques make it difficult to measure the BMD of vertical bone trabeculae alone. The T-scores, HU values, and VBQ scores are volume-based measurements of bone quality and are not directional. With the development of artificial intelligence, it may become a reality to identify the density of vertical bone trabeculae specifically through some algorithm in the future, so this may be a future development direction.

## Limitations

Our study has limitations. Firstly, the sample size of this study is not large enough, and the threshold obtained in this study needs to be verified by a larger sample study. Secondly, the study did not link the two imaging parameters with osteoporosis-related complications, which would have ignored special populations, such as patients without osteoporosis diagnosed by DXA who had an osteoporotic compression fracture. In addition, the correlation between the two parameters and osteoporosis-related complications still needs to be further studied. Thirdly, both HU values and VBQ scores are manually selected ROIs, and there is no uniform standard similar to QCT, so these can only be used as supplementary diagnostic tools for osteoporosis. Finally, as a retrospective study, we excluded patients based on exclusion criteria, so this may be biased against real-world performance, and prospective, more comprehensive data may be needed to verify this in the future.

## Conclusion

Firstly, both of the opportunistic imaging parameters could be used to assist in the diagnosis of osteoporosis, and the HU values were superior to VBQ scores in the evaluation of BMD. Secondly, different combinations of the HU values and VBQ scores could improve the specificity and sensitivity of the diagnosis of osteoporosis. Finally, these parameters at the single-segment level (LI HU value and L1 VBQ score) may be suitable for clinical use due to the same diagnostic performance as the average HU value and VBQ score and relatively simpler to measure.

## Data Availability

The datasets used and/or analysed during the current study available from the corresponding author on reasonable request.

## Abbreviations

CT: Computed tomography
HU: Hounsfield unit
VBQ: Vertebral bone quality
MRI: Magnetic resonance imaging
DXA: Dual energy x-ray absorptiometry
BMD: Bone mineral density
ROI: Regions of interest
SI: Signal intensity
CSF: Cerebrospinal fluid
